# A Next Generation Assets-Based Public Health Intervention Development Model: The Public as Innovators

**DOI:** 10.3389/fpubh.2018.00248

**Published:** 2018-09-04

**Authors:** Christiana von Hippel

**Affiliations:** Department of Social and Behavioral Sciences, Harvard T.H. Chan School of Public Health, Boston, MA, United States

**Keywords:** community health promotion, intervention design, user innovation, online platform, makerspace, positive deviance, assets based models

## Abstract

In the public health field, the design of interventions has long been considered to be the province of public health experts. In this paper, I explore an important complement to the traditional model: the design, prototyping, and implementation of innovative public health interventions by the public (users) themselves. These user interventions can then be incorporated by public health experts, who in turn design, support, and implement improvements and diffusion strategies as appropriate for the broader community. The context and support for this proposed new public health intervention development model builds upon user innovation theory, which has only recently begun to be applied to research and practice in medicine and provides a completely novel approach in the field of public health. User innovation is an assets-based model in which end users of a product, process, or service are the locus of innovation and often more likely than producers to develop the first prototypes of new approaches to problems facing them. This occurs because users often possess essential context-specific information about their needs paired with the motivation that comes from directly benefiting from any solutions they create. Product producers in a wide range of fields have, in turn, learned to profit from the strengths of these user innovators by supporting their grass-roots, leading-edge designs and field experiments in various ways. I explore the promise of integrating user-designed and prototyped health interventions into a new assets-based public health intervention development model. In this exploration, a wide range of lead user methods and positive deviance studies provide examples for identification of user innovation in populations, community platforms, and healthcare programs. I also propose action-oriented and assets-based next steps for user-centered public health research and practice to implement this new model. This approach will enable us to call upon the strengths of the communities we serve as we develop new methods and approaches to more efficiently and effectively intervene on the varied complex health problems they face.

## Introduction

Standard innovation practice in the public health field has traditionally been based on a deficit model that views members of at-risk populations as clients with unserved health needs. These individuals or communities are to be helped via expert-designed and -implemented interventions toward more healthful choices and outcomes. In this traditional model those receiving public health interventions are not themselves viewed as problem solvers or innovators of effective solutions. However, a movement toward an assets-based approach to intervention design and implementation has been growing in public health whereby applied assets-based intervention development processes emphasize activating and drawing on the strengths and skills of individuals, communities, and a through co-production of intervention programs and research (1, 2). Through focus groups, interviews, and the community advisory boards common to community-based participatory research, community members can be invited to provide useful inputs to public health experts. This input often includes context-specific knowledge of their pressing needs and the individual and community resources they have to draw upon in co-producing solutions. Yet, like public health's traditional approach to intervention development, this current version of an assets-based approach emphasizing co-production assumes individuals and community members themselves do not devise, prototype, and use innovations of their own devising prior to expert involvement. Innovation practices, however, are creatures of specific knowledge states and conditions. As these change, innovators—including public health innovators—can discover new opportunities for improved practices by looking toward the full range of assets possessed by individuals and the communities they serve: strengths, skills, and successful, existing solutions.

Recent research on product and service development innovation in the public household sector has revealed a major opportunity for a new assets-based approach to health innovation that goes beyond co-production: user innovation (3–5). It has been found, via nationally representative surveys in six nations (as described in detail later), that ~5% of citizens are not passive users—they actively innovate, developing new products and services to fulfill their own needs and gain the accompanying important benefits, completely independent of producer support. Therefore, user innovation refers to the development of novel products, services, and behavioral strategies by end users, such as communities or patients, without support from manufacturers or professionals. The innovations that household sector individuals develop include innovations to address their own medical and preventive health needs and significantly improve their quality of life, innovations that, in many cases, have been shared and found to be valuable to others as well.

In the business sector, leading-edge firms are using this discovery to develop and test new innovation models that complement their producer-centered, “find a need and fill it” models of innovation development. These new, “user-centered” models are based on systematic searches for user-developed solutions to their needs, rather than just for unmet user needs. Producers' confidence in this new approach is bolstered by historical studies [e.g., (3, 6)] showing that many of the most innovative products they sell—those that have pioneered new markets and applications—were, in fact, preceded by and often based upon innovation prototypes that users designed and built to serve their own needs. For example, people living with Type 1 diabetes have developed, tested, and are diffusing designs for artificial pancreases well ahead of commercial availability of such devices from medical device producers, thereby demonstrating the feasibility of incorporating user innovations into public health interventions for self-management of chronic disease (7).

In the body of this paper, I begin by reviewing recent research findings on users who develop new products and services to better serve their own needs and to benefit thereby—including almost a million who innovate to meet their own health needs—as well as their efforts to share and diffuse their innovations (section Health-Related Innovation by the Public/Users in the Household Sector). Next, I will outline a new assets-based model of public health intervention that incorporates the rich resources of health-related user innovation in the household sector (section An Assets-Based Public Health Intervention Model for the Next Generation) and then discuss a wide range of action-oriented and asset-based next steps to implement this new model (section Next Steps for Public Health Research and Practice Using an Assets-Based Intervention Development Process). These next steps will be based upon systematic searches for product and practice prototypes and solutions developed by public health users—individuals and community members—that have been found to be effective in the context of everyday life. Just as is the case in modern producer practice, these innovations can serve as useful prototypes in the model that can be assessed, improved, and, as appropriate, generally diffused by experts in the public health field as well as being a resource for research and improved practice.

## Health-related innovation by the public/users in the household sector

### The extent of new health-related product and service innovation by users

Nationally representative surveys, conducted in six nations to date, document that tens of millions of citizens have, within the last 3 years, developed new products to serve their own needs (Table 1). These six national surveys of household sector end users were restricted to product innovations only (excluding service and behavioral innovations). They were carried out in the United Kingdom (8), in the United States and Japan (9, 10), in Finland (11), in Canada (12), and in South Korea (13). As can also be seen in Table 1, the fraction of user innovation effort devoted to developing medical/health-related product innovations in the six national surveys varies by nation, ranging between 2 and 8% of all product innovation projects by users (Table 1). Given the large population of these nations, such a fraction represents an impressive total of almost 1 million individuals making medical innovations.

**Table 1:** Fraction and number of citizens^a^ developing household and medical solutions for their own use in six countries, amounting to almost 1 million patient innovators.

	**UK^b^ (*N* = 1,173)**	**US^c^ (*N* = 1,992)**	**Japan^c^ (*N* = 2,000)**	**Finland^d^ (*N* = 993)**	**Canada^e^ (*N* = 2,021)**	**S. Korea^f^ (*N* = 10,821)**
Percentage of user innovators in population	6.1%	5.2%	3.7%	5.4%	5.6%	1.5%
Number user innovators	2.9 million	16 million	4.7 million	0.17 million	1.6 million	0.54 million
Percentage of user innovations with medical purposes	2.0%	7.9%	2.4%	7.0%	8.0%	5.5%
Number of medical user innovators	58,000	384,000	371,300	11,900	128,000	29,700

*N refers to total survey sample size in each country*.

a *In all six surveys individuals under the age of 18 were excluded due to youth privacy considerations. Adults age 18 and older were included. No upper age limit was imposed except in the Finnish survey, which only included responses from adults ages 18–65*.

b *(8)*.

c *(9, 10)*.

d *(11)*.

e *(12)*.

f *(13)*.

This important fraction becomes much larger in research on samples that consist only of medical patients—individuals who actually have a medical need—and that expand the types of allowable innovations to include both products and medical self-services. Thus, a study of a sample of medical patients in Portugal shows a much higher fraction innovating to serve their own needs (53% vs. the typical range of 2–8%) (14). The 500 patients surveyed are coping with chronic diseases ranging from rare genetic disorders such as Angelman's Syndrome to more common but very serious chronic diseases like Type 1 diabetes. Of these 500 respondents, 53% reported developing a solution they viewed as novel. Almost all of their innovations were medical services rather than devices. Evaluation by medical experts found that 8% of reported innovations (40 of 500 respondents) had indeed developed innovations that were new to medical practice. The remainder had reinvented solutions that were not new to medical practice, but that were new to the respondents (re)developing them.

Patients also reported on average that their innovations significantly benefited them in managing their disease and improving their quality of life. For solutions that were new to the world, the improvement in quality of life of the patients was 2 points on a 7-point Likert scale ranging from 1 “awful” to 7 “excellent.” For solutions that were new to the patient but not new to the world, the mean improvement in patients' quality of life was 1.6 on the same 7-point scale. Caregivers reported mean improvements in the quality of their lives of 1.9 for novel solutions and 1.4 points for non-novel solutions. While some fraction of this quite large reported improvement was due to what is known as the “I designed it myself” effect (15) or the “Ikea effect” (16), these studies show that subjects greatly value and benefit from products and services that are self-designed or self-built to meet their needs.

For public health applications of the user innovation model, it will be important to understand how the intrinsic value of having self-developed their own health promoting solution may bias the community toward preferring a user innovation over an evidence-based intervention. Honoring the invaluable sense of accomplishment and personalization that comes from self-developing a product or program is a vital part of cultivating an assets-based approach to public health intervention development. Even in circumstances where it will take more time to achieve the desired health outcome, it may be both more effective and more sustainable long term to provide the community with tools for including and building solutions than for professionals to supply them with a ready-made, evidence-based intervention.

### Diffusion of health-related product and service innovations by users

When users develop an innovation and benefit by applying it to satisfy their own needs, findings in both Finland (11) and Canada (12) show about 80% are willing to have other users or commercializing firms discover what they have done and adopt it free of charge. However, because these individuals are giving the information away for free, they also have no inbuilt incentive to invest time or resources to actively inform others about their innovations or to help transfer their innovations to others (5). The net result is that only a fraction of potentially valuable innovations developed by users are in fact diffused beyond the originating individuals or groups. This situation is reflected in both the Finland national user innovation study data and also within the Portuguese patient study mentioned earlier.

In the case of data from representative surveys in six nations, the Finnish survey asked user innovators to rate their innovations in terms of likely general value (11). (Note that what is important to the issue of users investing effort in diffusion as a function of innovation general value is the users' own view of the value of their innovations, rather than some possibly more objective measure.) As can be seen in Table 2, perceived value to others greatly influences users' efforts to diffuse their innovations. Yet, the overall proportion of cases in which users invested effort to diffuse their innovations to peers or to producers is promising. It indicates that there may be an even greater desire to diffuse that could be facilitated into action if barriers to diffusion were removed.

**Table 2:** Diffusion effort across clusters of general value in Finland (*N* = 993).

**Perceived general value**	**Diffusion effort made by user innovators**	
	**To inform peers (%)**	**To inform producers (%)**
Cluster I: valuable to many	23	19
Cluster II: valuable to some	21	6
Cluster III: valuable to few	12	0

*de Jong et al. (11), table 6. Reproduced with authors' permission*.

The Oliveira et al. (14) Portuguese patient innovation study does not assess diffusion effort as a function of perceived general value of an innovation. However, it does report that a significant proportion of the 263 patients and caregivers who reported solutions, also reported investing efforts to share their solutions with others (32%, *n* = 84). Oliveira et al. asked about seven types of diffusion effort patients undertook (Table 3).

**Table 3:** Portuguese rare disease patients' solution sharing activities (*N* = 263).

**Solution sharing activities**	**Percent engaged in this activity (%)**
No effort devoted to sharing	68
Showed it to other patients	28
Showed it to medical professionals	2
Shared the info on a website/blog/social network	8
Shared it through media	2.25
Showed it to commercial entities	1
Spent time and/or money to help others (people, companies) use the solution	1.5
Made a manual or documentation that helps using the solution	1

*Oliveira et al. (14), table 5. Reproduced with authors' permission*.

The most common mode of sharing was patient-to-patient or peer-to-peer, reported by 28% of respondents (Table 3). Showing or describing an innovation to a medical provider occurred in only 2% of the sample. Actively diffusing information to commercial firms occurred in 1% of the sample. Similarly, 1.5% of patients and caregivers spent time or money to help diffuse their innovations and 1% also reported making a manual or documentation to help others use their solutions. As is reasonable, the nature or frequency of sharing effort engaged in by patients or caregivers did not significantly differ between new-to-the-world innovations and innovations that they only thought were new. The strongest predictor of information sharing across the sample was the magnitude of the improvement in quality of life patients felt from using their solution, assessed on a 7-point Likert scale ranging from 1 “awful” to 7 “excellent.” A 1-point increase in the improvement innovators felt their solutions generated increased the odds of sharing their solution by a factor of 1.7 (95% CI: 1.4–2.1). Note, however, that patient-developed innovations (2%) were seldom shared with medical professionals directly, and when they were, there was no further opportunity or means given for diffusion to or quality vetting by public health professionals. We will explore opportunities for identification, evaluation, incorporation, and further diffusion of these innovations by public health and medical professionals in the following sections.

## An assets-based public health intervention model for the next generation

**Figure 1:**
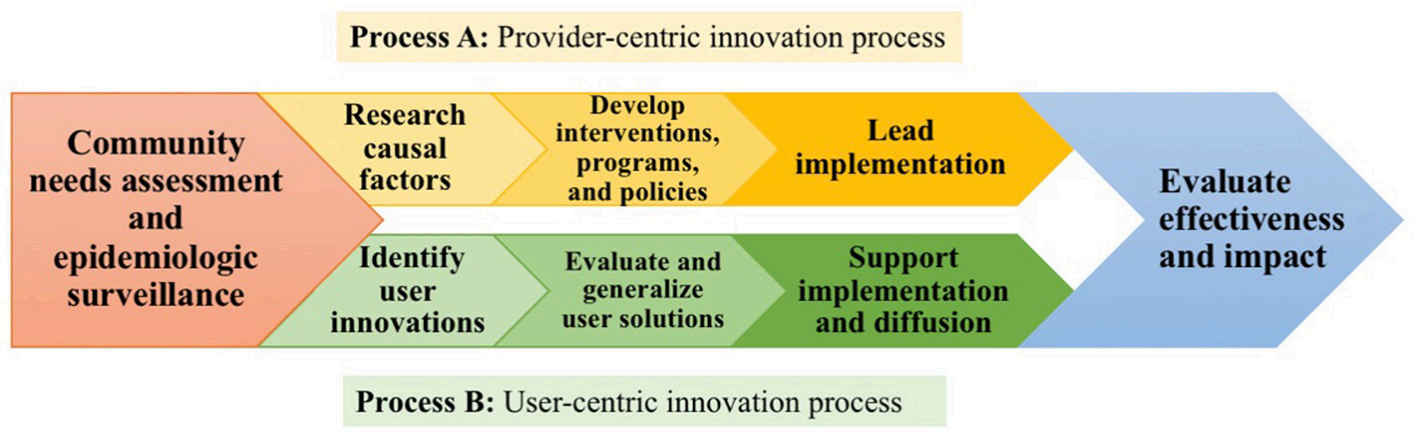
A model comparing the traditional “provider-centric” (Process A, yellow) and complementary “user-centric” (Process B, green) public health intervention development processes that have the potential to work together.

In light of the wide extent of health-related product and service innovation by the public in the household sector, I propose that the search for and assessment of unmet needs among communities and implementation of interventions that is traditional in public health research and practice (Process A in Figure 1) can be complemented by novel assets-based efforts to discover, evaluate, improve, implement, support, and diffuse public health innovations developed by individuals and community members themselves (Process B in Figure 1). These efforts can work together and complement each other, embedded in a new public health intervention model as shown in Figure 1.

As will be familiar to all public health scholars and professionals, the traditional public health intervention process begins with search by experts (e.g., epidemiologic surveillance) among populations for important, unmet public health needs. Generally, these are identified and quantified using broad population health surveys conducted by government agencies (e.g., CDC) and NGOs (e.g., WHO) to obtain morbidity and mortality data at global, national, and local levels. These data indicate the population distribution of each prevalent or emerging health issue. Public health experts in international, national, and local public health agencies then select the public health needs they will address. Within a chosen preventive health issue, the primary target population for a public health intervention is determined with respect to level of risk experienced by its members. In standard public health intervention design practice, after an at-risk population is identified for a given public health population, public health practitioners carry out research to better understand the need, themselves develop an intervention to address the need, then carry out the intervention, and, finally, assess the effects achieved, often through randomized controlled trials. Randomized controlled trials are often used to determine the true level of health improvement achieved by the intervention as compared to change that may arise from the passage of time or due to a standard intervention already offered to the community.

Note that Process B shown in Figure 1 differs from the traditional public health intervention process just described in several crucial aspects. As is the case with Process A, it begins by identifying a public health problem “worth going after.” Then, it diverges from Process A by seeking to identify innovations that have been developed by users within the community who are affected by the public health problem at issue and who have managed to *solve* the problem under real-world conditions. Public health experts then evaluate the solution(s) identified for safety, quality, and generalizability, improving the more promising ones as needed to fit the broader community context. Some solutions may, in fact, have already been evaluated and improved through community collaboration and replication via peer-to-peer implementation and diffusion. If the solutions have diffused within the community, diffusion strategies are also evaluated for promise as models of more general diffusion strategies public health experts could use in other contexts. At this stage, Process B frames the implementation stage as support for diffusion of user-developed solutions that have been refined by public health experts. Process B returns to the same pattern as Process A by concluding with an evaluation stage to assess the impact of the solutions on community health outcomes. Working together with the public as problem solvers through this complementary, assets-based intervention development process will allow the field access to a rich, untapped resource: innovative solutions already developed by users themselves.

## Next steps for public health research and practice using an assets-based intervention development process

We will next discuss three action-oriented, assets-based next steps to implement Process B of the proposed new public health intervention development model shown in Figure 1. These steps include: (1) identifying user innovations; (2) evaluating and generalizing user solutions; and (3) supporting implementation and diffusion of user solutions to broader populations.

### Step 1: identify user-developed health-related innovations

#### Lead user methods

Two general approaches have been developed for the discovery of user-developed innovations of possible interest to both peer adopters and producers of user goods or services. They are: (1) methods to identify and study “lead users” whom research has shown to be most likely to innovate in generally useful ways, and (2) methods to attract innovative users to reveal, co-develop, and share their innovations on web-based platforms or in makerspaces (i.e., workshops where members of the public with shared interests can gather to share knowledge, tools, and materials as they work on self-motivated projects) (see section Platforms for Identifying, Supporting, and Diffusing User-Developed Interventions below).

As we saw in section Diffusion of Health-Related Product and Service Innovations by Users above, via both studies of household sector innovation and medical patient innovation, a given population will contain some individuals who have developed solutions for themselves, as well as the many individuals with the unmet needs public health has traditionally focused upon. However, neither traditional public health “market” research approach to including community input in the research process or the less commonly used community-based participatory research approach (CBPR) (17) was designed to identify individuals with solutions. In the market research approach, conducting focus groups and interviews, often with convenience samples, provides insight into individuals' knowledge, attitudes, behaviors. It is unlikely, however, that user innovators will be represented in such samples or that the questionnaires used would elicit innovation or interventions that members of the at-risk public might have developed. Similarly, CBPR selects communities with pressing health needs and assembles an advisory board comprised of diverse community stakeholders who help decide on a specific research question and brainstorm solutions. CBPR is a valuable, assets-based approach, but one that emphasizes co-production at every stage of research, meaning that representatives of the community partner with researchers to design interventions that build upon their strengths and resources rather than working to elicit successful, existing problem solutions and interventions.

“Lead users” were first defined 30 years ago as individuals (or firms or organizations) that are: (1) at the leading edge of a commercially important trend in the market, and (2) who have a strong need to solve the novel problems that they encounter there, and so may innovate to solve them (6). Note that lead users are ahead of even “innovators” on standard innovation adoption curves (18). This is because they confront a situation where nothing suitable to their needs has yet been developed that they can adopt. It has been found that lead users who already possess technical development skills are the most likely innovators (19). It has also been found that the stronger the lead user characteristics of innovators, the more likely these individuals are to develop innovations of general commercial value (20). Still further, it has been found that user-generated product designs are rated significantly better than those developed by professional product developers in head-to-head comparisons (21, 22). Driven by these findings, many scholars have contributed to the development of methods for finding lead users, evaluating their innovations, and converting these prototypes into commercial products. As of 2008, 24% of US firms reported using the lead user method and finding it very effective (23).

Lead user methods are most useful when there are important trends that can be identified, relative to which lead users can be sought. The important downward trend in effectiveness of antibiotics provides an example of interest to the public health community. Lead user studies have shown, with respect to prevention of post-surgical infections, that surgeons in specialties at the leading edge with respect to the seriousness of such problems (e.g., oncologists dealing with patients whose immune systems have been compromised by chemotherapy) are also at the leading edge for developing solutions of general value (24).

Identifying lead users has radically become more efficient in recent years as user innovators increasingly organize collaborative innovation development and diffusion efforts via public websites. For example, lead user patients themselves have developed solutions to help them manage the chronic difficulties in day-to-day living associated with Type 1 and Type 2 diabetes. Those coping with conditions like Type 1 diabetes who also have technical skills have been very active in developing and freely sharing both behavioral interventions and technological solutions ahead of public health providers and medical equipment producers.

There is clearly an urgent public health need for such innovation: the standard approach to self-management of Type 1 diabetes requires a subtle and sophisticated balancing act of carbohydrate intake, physical activity, and insulin dosing to maintain blood glucose homeostasis. But among the 29 million people living with Type 1 diabetes in the US (25) there are roughly 100,000 emergency room visits related to insulin dosing every year (26), a growing health problem. The introduction of continuous glucose monitors (CGM) allows patients to more efficiently and more accurately maintain their blood glucose within recommended ranges compared with self-monitoring (27).

A group of adult patients and parents with children who have Type 1 diabetes recognized that while available continuous glucose monitoring (CGM) technology does allow patients to more effectively maintain their recommended blood glucose range (80–180 mg/dl) compared with self-monitoring, CGM devices had a significant deficiency. CGM alarms related to either hyper- or hypoglycemia could go unattended, especially while patients and caregivers sleep. In relatively short order, this group of parents, patients, and friends of patients collectively called “NightScout” managed to hack into the continuous glucose monitor software and provide for remote acknowledgment of either hyper- or hypo-glycemia by parents or trusted friends via smartphone. Thousands of patients with Type 1 diabetes use this patient-developed solution today. The open source code for it is distributed for free through NightScout's web platform and its 26,000-member patient community on Facebook called CGM in the Cloud. Preliminary evidence from a survey of 1,000 NightScout users shows that the majority experience fewer hyper- and hypo-glycemic episodes and report improved quality of life since adopting the remote monitoring system (7). Therefore, lead user methods can be used in public health to identify users on the leading edge of pressing health problems. As we have seen, an abundance of the innovations lead users develop out of necessity within their acute medical care contexts and in the day-to-day context of self-managing a chronic disease like diabetes can prove effective for the larger communities that surround them.

#### Platforms for identifying, supporting, and diffusing user-developed interventions

Firms have instituted some active search strategies for finding and supporting user innovations that they could integrate into their business. User innovation research findings showing that a wealth of ideas are being generated by users, but not necessarily diffused to their fullest potential, have prompted companies like Lego to explicitly support user innovation by housing it within their own online platform. Through the “Create and Share” platform within the Lego website (28) is essentially an online makerspace. Lego fans can comment on other users' designs and receive support to tinker with their own Lego bricks until they have built a design they want to share. By providing infrastructural support and encouragement to innovative customers Lego has engendered a synergistic relationship between fans and in-house research and development staff. Fans continue to buy the bricks and now enjoy building with them in a newly social way while Lego hosts the platform and gains potentially lucrative insight into their customers' design preferences (29, 30).

In the context of health where there is often no firm to provide the infrastructure, individuals with the same health concern may create collaboration platforms for themselves. In the case of Type 1 diabetes user innovation has flourished through collaboration on the NightScout peer-to-peer internet platform described earlier. NightScout has eased the creation and diffusion processes challenges that user innovators often face. They have done so by creating a community where the collaborative model of problem solving lowers the barrier to sharing information and engenders social cohesion. This platform is known to a growing number of health professionals because diverse members themselves have broadcasted their activities through social media. Therefore, a search method for user-developed innovation platforms related to other health issues could be to look for the clusters of online activity around problem solving within patient support websites, do-it-yourself (DIY) project discussion boards, etc., rather than for the problem solvers themselves (i.e., Nightscout was not found by looking for diabetics with technical skills or other particular markers of innovation potential).

Exactly this approach was used to form Patient-Innovation.com (31). This platform was developed at Católica Lisbon School of Business and Economics in 2014 with sponsorship from interdisciplinary partners that include multiple Portuguese medical and research organizations. The founders initially seeded the site with user-developed solutions that they found posted within more general problem-solving websites such as Thingiverse and Instructables as well as on social media (e.g., Facebook, Instagram). They looked for postings of DIY solutions designed by patients with rare and chronic diseases to improve their quality of life that got likes, views, and comments from other users.

The Patient Innovation platform illustrates a unique example of academic and clinical support for the diffusion of innovations developed by patients that is gathering momentum—and that the public health field can easily support. The platform engages patients and caregivers in searching, posting, and discussing user-developed solutions that improve their quality of life with rare and chronic diseases. Of the 700 solutions currently listed on the website, one example comes from a breast cancer survivor who posted her solution for the challenge keeping her mastectomy surgical drains dry while bathing the rest of her body. The breast cancer survivor's “shower shirt” is a comfortable, water-resistant, reusable remedy for covering the chest that replaced the use of plastic wrap and trash bags that patients were used to struggling with on a daily basis during their recovery. She has since patented this solution and patients with other conditions requiring protection of the chest from water (e.g., after rotator cuff or cardiac surgery) have since adopted it as well.

#### Positive deviance methods

A different assets-based approach that has been deployed in the public health field specifically to identify and build upon solutions developed by individuals and communities themselves is known as Positive Deviance (PD) [postulated by (32) and formalized by (33)]. The public health field has some experience with PD methods (34–38). These examples of the PD approach to public health are in line with the general direction of lead user methods. Their reported success strengthens my confidence in the new directions for public health interventions I am proposing here.

The PD approach is based on the observation that in many communities a few members exhibit uncommon but beneficial behaviors that enable them to find more effective solutions to community problems. These positive deviants achieve better outcomes than their peers despite facing similar challenges and residing in contexts where they have resources similar to those available to their less successful peers (39). If for example, one has the public health goal of helping a community in the developing world to improve child health, the basic positive deviance study approach [e.g., (40, 41)] would be to search for families in the community who stand out as positive deviants with respect to good developmental outcomes. The assumption is that such families or individuals within them have somehow developed or found a solution that is useable in their community context and successful for them. Researchers then seek to understand what these deviants are doing that is linked to better health. Then, if that solution looks viable for others, they may attempt to diffuse it in the community [cf. (42)].

In the seminal case example of PD at work, the US NGO Save the Children sought a solution to Vietnamese child mortality from malnutrition other than sending in nutritional supplements from outside. Mothers in the community with well-fed children had figured out two solutions. First, they added tiny shrimp and crabs from the local rice paddies in their family's communal soup cooking pot even though these were considered inappropriate foods for children. Second, at mealtime they had taken to feeding their children first from the protein-rich bottom of the pot rather than last when only broth was left. Once communicated to the rest of the mothers in the community as socially normative, this user-developed behavioral intervention reduced Vietnamese childhood malnutrition by 80% (43).

The major drawback to the positive deviance approach is primarily the time and expert effort required to implement it. Prescribed practice involves researchers embedding themselves within a community in order to discover examples of positive, uncommon strategies. These are costly to identify at a prevalence of 1–10% (44). In essence, the lead user approach solves these practical limitations in the PD approach by utilizing improved tools for identifying valuable positive deviants both within and outside of any given community. It incorporates the understanding that those with more intense needs are more likely to develop solutions to a given problem, and so efficiently searches for “high-need, high-benefit” individuals across an entire nation or the entire world, using tools described earlier.

#### Building trust and tools to discover innovations within marginalized communities

In the case of public health problems that are stigmatized within a population, innovators may not be willing to risk revealing their behavior by sharing their solutions widely. For example, in the Vietnamese study discussed just above, it was found that mothers in the Vietnam study did not speak outside the home of their use of protein-rich soup to supplement their children's nutrition because feeding shrimp and crabs to young children was against their community's custom; they could be ostracized by other mothers for doing so (43). Similarly, fear of social stigma may have deterred many Portuguese medical patients from sharing their innovations with their doctors. Patients said this omission is due in part to their doctors' perceived lack of time and interest to hear about their innovations compounded by fear of being judged by a clinical authority figure or labeled “noncompliant” (14).

Still, there is evidence that, by building trust, something public health is experienced at, innovations can also be discovered among marginalized groups like intravenous drug users (IDUs). Consider that IDUs live with multiple health threats that necessitate independent problem solving: HIV/STI transmission, social stigma, legal prosecution, and the consequences of over or under dosing (withdrawal). Given the sensitivity of this group's behavior and health needs, one might assume it would be difficult to identify a sample of positive deviant lead users and engage them in a study. But one study (36) did so successfully by scanning New York City's records of intake assessments at needle exchanges and drug treatment facilities providing services to long-term drug users to find and recruit both participants who had multiple negative HIV and Hepatitis C (HPC) tests and those who had positive test results for one or both viruses. They then conducted a study to uncover the HPC/HIV prevention innovations that these long-term IDUs had developed over time.

Friedman et al. (36) were guided by grounded theory in collecting IDUs' life histories over the course of multiple exploratory interviews. Of the 35 long-term IDUs they interviewed some had contracted HPC or HIV or both from tainted injection equipment or unprotected sex with another drug user. But the majority had managed to use for over 10 years without contracting either HPC or HIV. These individuals did not stay safe simply by exchanging used needles for free, clean ones at local nonprofits as public health professionals would advise. They disclosed social and behavioral innovations—termed “strategies” or “tactics” by IDUs—for remaining uninfected ranging from sniffing drugs rather than injecting them when clean equipment was not available to only injecting alone or with a small group of trusted peers. These peers would presumably not pressure them to use too much or with shared equipment when their judgment was compromised by intoxication.

Driven by their incentive to both remain disease free and continue using drugs, IDUs innovated these successful harm reduction techniques that public health professionals would likely not have devised. As a consequence, the discovery of lead users, the positive deviants who have both displayed an incentive to solve their problem and have successfully solved it, will provide public health researchers with information on both incentives that can motivate public health users and solutions that can be successfully applied and promoted by public health experts. The lead user definition and approaches discussed earlier in this paper could enhance the quality of community-generated approaches still further, and also render the collection of the needed information much more efficient. For example, following the lead user logic and methodologies, one would focus one's positive deviance research efforts on successful innovation among IDU's facing conditions of extreme need—such as those in the context of a HPC outbreak—rather than upon convenience samples.

Public health researchers are also well positioned to discover innovations among another hard-to-reach group—women. In the context of user innovation research, women's innovations have historically been difficult to capture, perhaps due to a gender bias in innovation research. But the driver of this gender discrepancy may be the user innovation field's emphasis on product innovations. Product innovations are developed most often by technically skilled individuals with emphasis on software programs and engineering hacks that are less prevalent among women in general (8). Behavioral innovations (sometimes referred to as “creative coping methods” for ease of understanding by the public) may be more common among women, especially if conceptualized as being resourceful in the face of everyday life challenges. But it will be necessary to develop innovation measures specifically designed to capture behavioral innovations and to validate them among communities of women (e.g., breast cancer patients coping with persistent quality of life issues following treatment, mothers of children with special health needs, etc.). This would significantly advance the literature on user innovation. Even more importantly, in the public health field, it could potentially reveal a number of generalizable coping methods that, by their nature of being behavioral changes rather than products, may be low-cost, simple to implement, and rewarding—thereby making them accessible even to resource-poor communities.

#### Partnering with healthcare practitioners as public health innovators and diffusers

Behavioral innovations and other low-effort, high-impact innovations of value to public health can also be found among medical professionals. The Veterans Administration Healthcare System in Pittsburgh had great success in 2005 when they used the positive deviance approach to find hundreds of small solutions that worked together cut their rate of hospital acquired methicillin-resistant Staphylococcus aureus (MRSA) infection in half (45, 46). The VA could not afford to swab test and decolonize every MRSA carrier upon entry into the hospital. Instead, clinicians and administrators worked together to identify the innovations for improving hand hygiene and environmental (e.g., hospital surfaces, medical devices) disinfection that were being used in the hospital units with the least burden of infection.

One of these innovations was a nurse's practice of pressing bacteria ridden elevator buttons with only a knuckle because she discovered it is an area of the hand less likely than the fingertip to transmit bacteria to the next object it touches. Even small changes like this can have a powerful cumulative impact. Other solutions were device alterations such as wrapping patient monitor screens and keyboards with two layers of plastic wrap—the first to prevent fluids and bacteria from getting onto the device and the second to prevent bacteria from being transmitted between users of the devices. This outer layer of plastic would be torn off and replaced after each use.

Communicating knowledge of internally developed user innovations between hospital units helped engender a culture of collaborative innovation at all levels of the hospital staff and sustained the control of MRSA incidence at the VA even after the Positive Deviance study period had concluded (45, 46). Following the logic of the lead user research approach, one could also explore methods of avoiding bacterial contamination at cancer centers, which have an extreme need to protect immuno-compromised patients from infection as they undergo chemotherapy. Another source could be hospitals in very difficult contexts like resource poor medical environments in developing countries that nonetheless have some of the lowest rates of hospital-transferred infections among their patient populations.

### Step 2: evaluate and generalize user solutions

In all of the above ways of identifying health-related innovations by users in the household sector, evaluation and generalization take place during the course of devising, prototyping, and implementing solutions to problems. Clinicians and staff at the Pittsburgh Veterans Administration Healthcare System, for example, collaborated in their positive deviance approach to reducing the rate of MRSA infection, tracking infection rates for each hospital unit, comparing disinfection techniques in units with high and low MRSA rates, and iteratively implementing new techniques that made measurable improvements. Their evaluation process was embedded within their search for innovations and comparing across hospital units throughout the process contributed to the ultimate generalizability of successful MRSA prevention practices.

### Step 3: support implementation and diffusion

#### Platforms and makerspaces

As we saw with Patient-Innovation.com, Lego, and NightScout's CGM in the Cloud Facebook group, web-based platforms help identify, support, and diffuse user innovations. Online diffusion of user innovations encourages large-scale communities of otherwise disparate users to form and provides communication tools that empower members to continue to improve upon solutions to shared problems. To encourage a social norm of iterative innovation off-line as well, medical makerspaces have recently begun to be installed within hospitals and clinics. Clinicians constantly modify and develop new devices to improve patient outcomes and make their own jobs safer or more efficient—often improvising to address the need at hand. Solutions range from modifying available, child-sized bandages to fit a newborn, to developing an openable plastic cast that is less cumbersome to apply to a broken limb than plaster (47, 48).

Busy clinicians, however, often make little effort to diffuse their solutions, missing the opportunity for others with similar on-the-job challenges to evaluate and adopt them. In response, MakerHealth™ Spaces have embedded user innovation into the healthcare delivery process at American, academic medical centers like the University of Texas Medical Branch (49). VINNOVA (The Swedish Innovation Agency) has also built several makerspaces just outside of clinics, in hospital administration offices where space is at less of a premium (48).

Access to workshops equipped with tools like 3D printers, sewing machines, glues etc. and peers with whom to develop ideas has successfully enabled doctors and nurses to improve their innovations and build new ones—often in collaboration with patients. These centralized innovation hubs have eliminated barriers to diffusion by building a sense of community around “making” and rewarding innovators with recognition of the contribution their ingenuity makes to patient care. Likewise, they have enabled researchers and hospital IRBs to evaluate emerging innovations for efficacy, safety, and potential generalizability to mainstream practice. These medical makerspaces as well as those more general makerspaces proliferating at the community level (e.g., in libraries, schools, and community centers) could be excellent sources of intervention designs for public health practitioners to explore. As evidence for the health protective value of makerspaces continues to grow, public health researchers will also have a valuable role to play in determining where and how to organize new ones. This effort will likely call upon our field's expertise in advocacy to support the expansion of makerspaces oriented toward inspiring innovations that serve population wellbeing, not just patient care. Through related policy initiatives, we will also be able to support equal access to makerspaces. Diverse community members have an invaluable problem solving perspective to offer and should be guaranteed access to makerspaces regardless of their gender, race/ethnicity, socioeconomic status, or healthcare plan.

## Conclusion

In this paper I have described the possibility and promise of complementing the traditional, expert-driven model of public health intervention development with an assets-based health intervention model that builds upon innovations developed by members of the public. However, building systematic practices and mechanisms to identify, evaluate, support, and diffuse public health interventions devised and prototyped by the public is a new challenge for the public health field. As was discussed, both communities of individuals suffering from public health problems and firms in the private sector have already led the way, and our field can learn from and build upon what has been done, expanding our own existing assets-based methods along the way. There is much evidence to support that health innovations developed by individuals to solve their personal problems can be renewed and repurposed as public health interventions through the user-centric process I have proposed in our assets-based public health intervention development model (Figure 1). These individuals may be medical patients and caregivers as seen in the case of NightScout or they may be community members who face preventive health issues such as poverty and child malnutrition as seen in the Vietnamese positive deviance study (43). As indicated in the model, the first step for public health will be to establish effective methods for identifying and supporting user innovators, methods and approaches that have been discussed in this paper.

Public health is well positioned as a field to provide diffusion support to user innovators. Health communication research in our field can identify barriers to diffusion to peers and directly to health professionals. It will also inform approaches to improving public understanding the meaning of user innovation and how it manifests in their communities. Improving users' knowledge that they are innovating can be a first step toward activating them to perceive their innovations as valuable contributions to the community and begin to diffuse. Simultaneously improving online diffusion platforms so that users are motivated to contribute their innovations through recognition and reward mechanisms will call upon the skill of public health's behavioral economists and experts in the psychosocial determinants of health behavior. In the field of consumer and industrial product development a similar model has proven its efficiency and effectiveness. I invite others to join me in exploring and developing the applicability of user innovation insights to the public health field, with the goal of more efficiently and effectively intervening on challenges to population well-being.

## Author contributions

The author confirms being the sole contributor of this work and approved it for publication.

### Conflict of interest statement

The author declares that the research was conducted in the absence of any commercial or financial relationships that could be construed as a potential conflict of interest.
